# Approach to Optimization of FRAP Methodology for Studies Based on Selected Monoterpenes

**DOI:** 10.3390/molecules25225267

**Published:** 2020-11-12

**Authors:** Karolina A. Wojtunik-Kulesza

**Affiliations:** Department of Inorganic Chemistry, Medical University of Lublin, 20-059 Lublin, Poland; karolina.wojtunik@umlub.pl

**Keywords:** FRAP, terpenes, antioxidant

## Abstract

Terpenes, wide-spread secondary plant metabolites, constitute important parts of many natural compounds that hold various biological activities, including antioxidant, calming, antiviral, and analgesic activities. Due to their high volatility and low solubility in water, studies of compounds based on terpenes are difficult, and methodologies must be adjusted to their specific characteristics. Considering the significant influence of iron ions on dementia development, the activity of terpenes in reducing Fe^3+^ represents an important area to be determined. Previously obtained results were unreliable because ferric-reducing antioxidant power (FRAP) methodology was not adjusted regarding studying terpenes. Taking this fact into account, the aim of this study was to optimize the method for monoterpene assessment. The study included three modifications, namely, (1) slightly adjusting the entire FRAP procedure, (2) replacing methanol with other solvents (heptane, butanone, or ethyl acetate), and (3) adding Tween 20. Additionally, a thin layer chromatography (TLC) -FRAP assay was performed. The obtained results revealed significant improvement in the reduction activity of selected terpenes (linalool, α-phellandrene, and α-terpinene) in studies with Tween 20, whereas replacing methanol with other solvents did not show the expected effects.

## 1. Introduction

The antioxidant effect is a well-studied, natural-compound bioactivity which can be determined by numerous methods, including spectrophotometry, chromatography, and theoretical consideration to account for in vitro and in vivo conditions [1,2]. Assays are based on scavenging free radicals (e.g., hydroxyl, superoxide), performing typical reduction reactions (e.g., reduction Fe^3+^ to Fe^2+^), or inhibiting pro-oxidant enzymes (e.g., xantine oxydase), etc.

From a medical point of view, Fe^3+^ plays important roles in several harmful oxidation processes within the human organism. This results from the fact that the ion is responsible for the aggregation of hyperphosphorylated tau, and is associated with neurofibrillary tangles, as well as progressive supranuclear palsy (PSP). Reduction of Fe^3+^ to Fe^2+^ can reverse this process and solubilize tau species characteristic of neurodegeneration [3]. Among methods used to determine the reduction ability of Fe^3+^ to Fe^2+^ is ferric-reducing antioxidant power (FRAP), a colorimetric method which uses the ability of antioxidants to reduce the colorless [Fe^3+^-(2,4,6-Tris(2-pirydyl)-*s*-triazine)_2_]^3+^ complex to the intensively blue-colored complex [Fe^2+^-(TPTZ)_2_]^2+^ in acidic medium [4]. Such color changes are spectrophotometrically measured at 593 nm. Results are calculated based on using ferrous ion standard solution and certain antioxidant standards (in most cases, trolox). The outcome is considered in FRAP units, where one FRAP unit can be defined as the reduction of 1 M ferric ion to one ferrous ion [5]. This method is widely used to determine the antioxidant activity of plants, foods extracts, biological fluids, spices, vegetables, fruits, and many extracts and essential oils.

A FRAP assay requires specific conditions, including, among others, an acidic medium (pH 3.6) to facilitate iron solubility and a temperature of 37 °C. The low pH decreases the ionization potential that drives electron transfer and increases the redox potential, causing a shift in the dominant reaction mechanism [6,7]. Similarly to other assays, FRAP has limitations. The most important is the redox potential of the pair Fe^3+^/Fe^2+^, because any compound with redox potential lower than this can induce falsely high Fe^3+^ reduction results. Additionally, FRAP assay results depend on the timescale of analysis. The assay is based on the assumption that the redox reaction proceeds rapidly and that all reactions are complete between four and six minutes. This is not always the case [7].

An important feature to be considered at this point is the pro-oxidant character of the assay. Research established that one of the reaction products is Fe^2+^, a pro-oxidant molecule that takes part in a Fenton reaction, leading to the formation of a hydroxyl radical. Nevertheless, numerous scientists explained that this problem can be resolved automatically in the reaction environment by using highly active antioxidants (e.g., polyphenols), which, despite activity toward Fe^3+^, reveal an ability to scavenge free radicals [8].

The described method is widely used in biological activity studies. At this point, it is worth stating that the reaction environment is hydrophilic; an acetate buffer serves as base for the reaction, while HCl solution is applied to prepare the TPTZ solution and water is supplied for the Fe^3+^ solution. The environment drives a situation wherein the reduction activity of tested compounds largely depends on their solubility in the measured mixture. The issue of an aqueous reaction environment is not a problem in the case of alcoholic extracts because of their high solubility in water. The problem appears when dealing with hydrophobic compounds (e.g., terpenes), which are hydrocarbons and derivatives of hydrocarbons with low hydrosolubility.

The aim of this study was to evaluate diverse FRAP reaction conditions (e.g., Tween 20 addition, replacement of methanol with solvents of various polarity) in order to optimize a method for application with a selected group of terpenes (γ-terpinene, citral, citronellal, carvone, α-phellandrene, α-terpinene, α-pinene, farnesene, eucalyptol, terpinene-4-ol, β-myrcene, p-cymene, linalool, β-myrcene, isopulegol, menthol). An additional aim of this study was to ascertain the effect of combining FRAP with thin-layer chromatography (TLC). This is pioneering work, as previous literature data did not provide information about the TLC-FRAP method. The presented study is based on assessing terpenes possessing numerous biological activities, including antioxidant, acetylcholinesterase (AChE) inhibitory, anti-inflammatory, sedative, analgesic, etc. [9,10]. Among the tested compounds were terpenes diverse in terms of structure and chemical properties (e.g., ketone–carvone, alcohol–menthol, and hydrocarbon–farnesene).

## 2. Results

### 2.1. Spectrophotometric Studies

**Figure 1:**
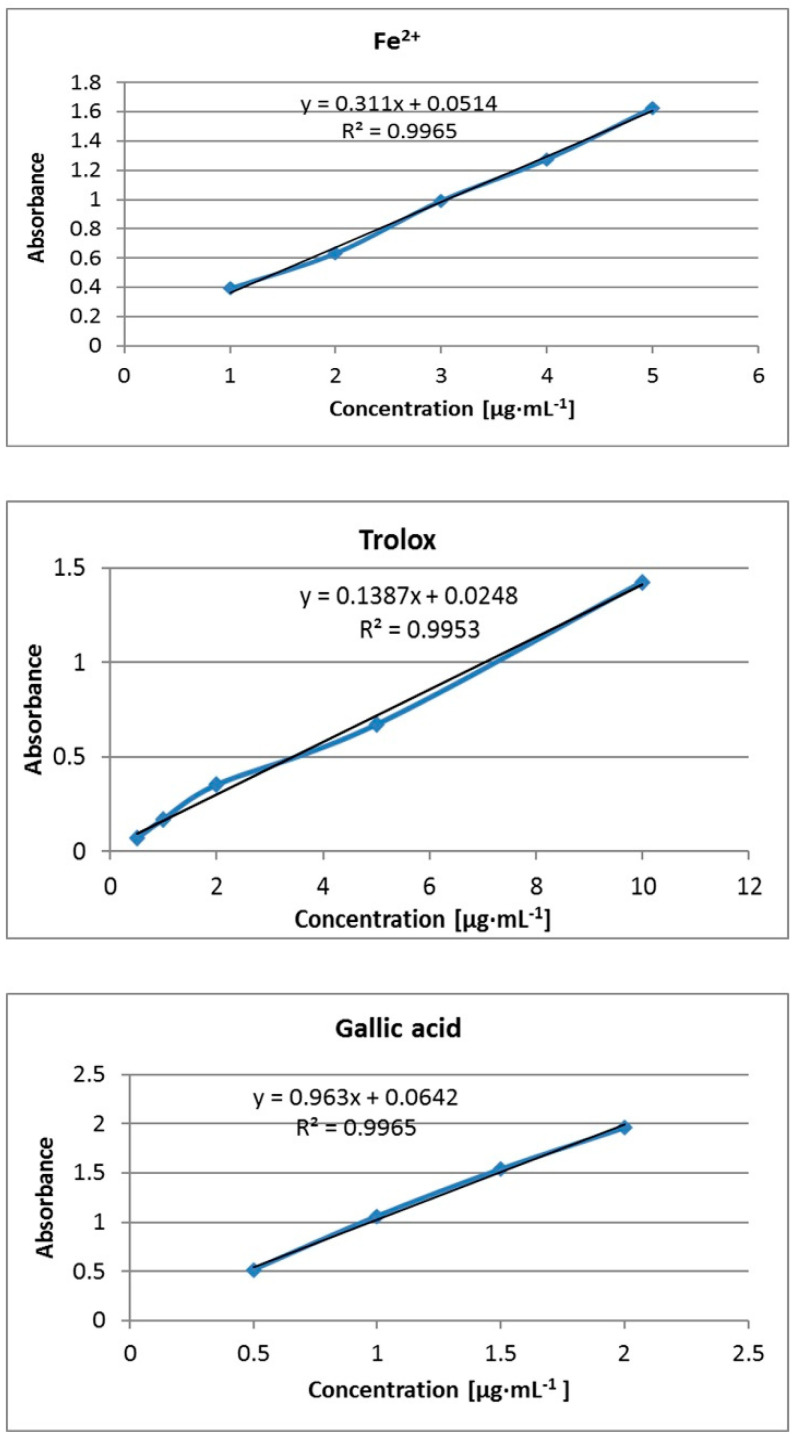
Calibration curves prepared for Fe^2+^, trolox, and gallic acid.

The first step of the performed studies was to ascertain the effect of only slightly modifying the basic methodology [11]. This involved replacing part of the water component with methanol to better dissolve the terpene content. This approach appeared to be ineffective. First, measurements based on the aqueous reaction environment caused clouding, in many cases without the combination of the FRAP solution with the studied terpene and, as a consequence, false-positive assay results were obtained. For the most part, the blue color as the result of [Fe^2+^-(TPTZ)_2_]^2+^ creation was not observed and the clouding induced an increase in absorbance value. A slight modification based on terpene dilution in methanol before combination with FRAP solution, however, led to a positive effect. A 0.1 M terpene concentration revealed high citral, carvone, and γ-terpinene activity levels, and the reaction progression showed a deepening blue color without clouding (Table 1). In the case of α-phellandrene and α-terpinene, the blue color was also observed but alongside the appearance of clouding. In this case, the results were considered to be false-positive. The remaining terpenes, namely terpinene-4-ol, linalool, p-cymene, β-myrcene, citronellal, isopulegol, menthol, menthone, farnesene, and eucalyptol did not exhibit Fe^3+^ reduction activity, with no blue color and observation of clouding. The active three terpenes were analyzed in detail, with the activity considered in FRAP units, trolox, and gallic acid equivalents (Figure 1).

The second step of the experiment intended to modify the method to enhance terpene solubility and avoid clouding. One possible way of obtaining a clear, non-clouded solution is to supplant methanol with another solvent. Taking into account that previous studies [12] revealed the positive influence of chloroform, ethyl acetate, and 2-butanone on the antioxidant activity of the selected group of terpenes, these solvents were applied in the FRAP assay. The basis of this modification was to replace the methanol component (used to dissolve the terpenes) with the three listed organic solvents. However, this minor modification did not bring the expected improvement in quality of the studied solution. In most cases, the clouding was stronger, probably resulting from high differences in polarity between the water/acetate buffer and the organic solvents. Hence, while the terpene solvents are effective, when added to the FRAP solution the outcome was extensive clouding.

In order to obtain a clear solution and reliable assay results, the addition of the surfactant Tween 20 to standard FRAP solution was investigated. This particular surfactant was chosen because it is characterized by very low toxicity, and is therefore often used in various branches of chemistry and pharmacy [13]. The outcome of this experiment was that, in a few cases, Tween 20 addition improved the solubility and study potential of certain investigated terpenes, as indicated by the appearance of the blue color and the reduction or disappearance of clouding. Among these were isopulegol, linalool, α-phellandrene, and α-terpinene (for which clouding was observed previously). Unfortunately, in many cases, Tween 20 augmentation resulted in clouding. These results are presented in Table 2. 

The obtained results revealed that Tween 20 addition influenced the reduction activity of the analyzed terpenes. As indicated in Table 1 and Table 2, the figures for FRAP units, trolox equivalent and gallic acid equivalents were reduced with regard to γ-terpinene, α-terpinene, citral, carvone, and α-phellandrene after assay modification when compared to the unmodified assay. In contrast, isopulegol, linalool (for all factors), carvone (0.5 mg·mL^−1^) (for gallic acid equivalent), and α-phellandrene (1 mg/mL) (for FRAP units) exhibited higher reduction activity for the FRAP/surfactant solution. The remaining group of studied terpenes did not reveal a positive response after the modification. A slight difference was observed, however, α-pinene exhibited color appearance, albeit with simultaneously clouding.

### 2.2. TLC–FRAP Assay

**Figure 2:**
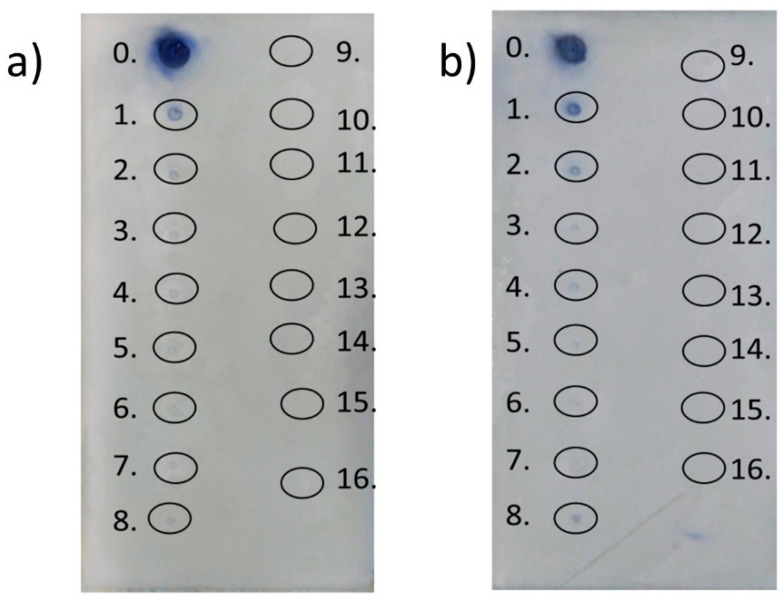
Results obtained for TLC–FRAP assay: (**a**) FRAP assay without modification; (**b**) FRAP with Tween 20. Active compounds appeared as dark spots on the light background. The compounds are numbered as follows: 0—gallic acid; 1—p-cymene; 2—eucalyptol; 3—farnesene; 4—carvone; 5—pulegone; 6—citronellal; 7—terpinene-4-ol; 8—γ-terpinene; 9—citral; 10—α-phellandrene; 11—menthone; 12—α-pinene; 13—linalool; 14—α-terpinene; 15—β-myrcene; 16—isopulegol.

The approach to combining thin layer chromatography with FRAP assay required some small modification of the originally intended method. The first attempt to accomplish this was based on the basic methodology with Tween 20 augmentation at standard ratio, but the obtained results were not satisfactory. In order to improve the method, the ratio of the FRAP solution component was changed to 10:2:2. This improved spot contrast and facilitated results analysis. The obtained results are presented in Figure 2.

The obtained results revealed a slightly positive impact of Tween 20 addition on terpene activity. When comparing the two TLC plates, the surfactant augmentation brought about a situation wherein p-cymene, eucalyptol, and γ-terpinene exhibited better reducing activity, although much less than gallic acid, a standard antioxidant. None of the remaining terpenes showed reduction activity in the proposed test.

## 3. Discussion

One possible way of counteracting the action of free radicals is to reduce trace metal ions able to generate reactive forms. Previous papers, however, presented few methods based on metal ion reduction. The reduction process in our organism is important because of its contribution to neurodegeneration [3,14]. Among the methods to investigate this reduction effect is FRAP, which is based on reducing Fe^3+^ to Fe^2+^. An important limitation of the FRAP assay is the fact that it is based on an aqueous solution (acetate buffer), hence, the method is limited to dealing with hydrophilic substances, whereas plant essential oils and their antioxidant constituents (i.e., terpenes) are hydrophobic. Considering this, modification of the method is essential.

As mentioned previously, the presented studies that researched the antioxidant properties of terpenes revolved around investigating the effect of modifying the standard FRAP method by adding methanol (in order to resolve the analyzed terpenes) and then replacing methanol with various solvents, as well as augmenting the basic FRAP recipe with surfactant Tween 20. The results are discussed below.

The first modification, a methanol–terpene mixture, showed a positive influence in comparison to standard procedure (adding the analyzed compound to the standard FRAP mixture of acetate buffer, FeCl_3_ in distilled water and TPTZ in HCl). The unmodified assay could not be undertaken due to the very low solubility of terpenes in the prepared solution. Regarding standard FRAP procedure, in almost all samples, clouding and/or insoluble substances were observed. The FRAP and methanol combination significantly improved the solubility and allowed determination of the antioxidant activity of the analyzed secondary plant metabolites.

In order to improve the solubility, the methanol component was replaced by various solvents that previous studies (details in [12]) found positively influenced terpene antioxidant activity. Among these were ethyl acetate and chloroform. While these are good terpene solvents, they negatively influence the prepared FRAP solution due to weak ability to mix with the aqueous solution. 

One possible way to improve solubility is surfactant addition. All surfactants are characterized by displaying critical micelle concentration (CMC), the concentration above which surfactants can form micelles and aggregate with solutes, potentially leading to changes in their observed activity [15]. In the case of this type of modification, the CMC criteria must be considered because micelle formation can lead to changes in obtained results. In our work, in order to avert this phenomenon, the surfactant concentration was below the CMC. As shown in Table 1 and Table 2, both positive and negative influences of Tween 20 addition were observed. A positive influence was noted for isopulegol and linalool, as their solubility improved in the studied solution. The other terpenes showed weaker reducing activity after Tween 20 addition. Analysis of the obtained results indicated that the differences may have resulted from the low solubility of substances in the nonmodified assay. Despite the solutions not appearing cloudy to the naked eye, detailed spectrophotometry analysis registered slight clouding, thus higher absorbance and false-positive results. Considering the positive influence of surfactants on solubility, as well as adherence to the CMC value, the reduction of biological activity of the studied compounds was not considered to be linked with the impact of surfactant on micelle creation. This fact was shown by two of the analyzed terpenes, (isopulegol and linalool), which revealed higher activity with surfactant addition, observed as blue color appearance as opposed to white clouding, as demonstrated in the previous stage of the experiment. The positive influence of Tween 20 addition was also observed for α-terpinene, which revealed a false-positive effect in the non-surfactant assay, while the activity significantly increased with a concentration of 0.1 M after surfactant addition. In this case, absorbance measurement was possible for smaller concentrations equal to 0.1 mg·mL^−1^, whereas for 0.1 M, absorbance measurement was impossible due to too high absorbance resulting from the deep blue color of the sample.

**Figure 3:**
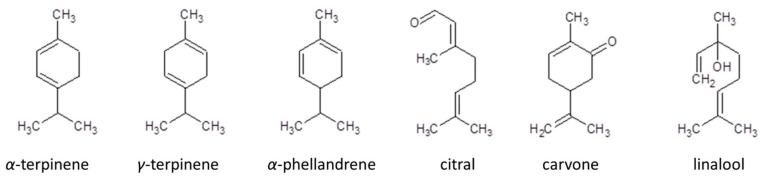
Structures of the most active terpenes in assay modified by Tween 20 addition.

Detailed analysis of the obtained results revealed some structure–antioxidant activity relationships related to certain antioxidant mechanisms. In accordance with Sadeer et al. [16], FRAP is based on an electron transfer mechanism in which aryloxyl radicals are formed. In the presented studies, the highest reduction activity was observed for cyclic hydrocarbon terpenes with conjugated double bonds without single electron transfer (SET) moiety characteristics (with the exception of γ-terpinene without the moiety) (Figure 3).

In accordance with Spiegel et al. [17], lower FRAP assay activity was observed for methylated compounds compared to their nonmethylated counterparts. The authors explained this phenomenon by stating that the methylation process decreases the activity of electron- and hydrogen-donating groups. Simultaneously, they noticed that along with decreasing distance between carboxylic groups and ring formation, the influence of methylation increased. In a study of the terpene structure, it was noticeable that the most active did not include moieties characteristic for the single electron transfer (SET) mechanism (e.g., –OH). This effect could signal higher activity in relation to the remaining compounds. In our work, only citral, carvone, and linalool contained moieties, however, these compounds turned out to be weaker Fe^3+^ reducing agents. This phenomenon could be explained by the presence of conjugated double bonds and greater bond dissociation energy for allylic, alkylic, and vinylic hydrogen atoms. In accordance with [12], similar studies were obtained for reactions with 2,2-diphenyl-1-picrylhydrazyl (DPPH). While the reaction mechanism was different, the obtained results revealed explicit dependency between the terpene conjugated double bonds and their ability to reduce Fe(III). In our work, the high activity of γ-terpinene was noted. Despite lacking conjugated double bonds, γ-terpinene showed high reducing activity resulting from the resonance stabilization of the compound. Hence, in accordance with [18], allylic hydrogen transference from the discussed terpenes leads to resonance-stabilized radicals that are able to terminate the chain reaction. Considering terpene activity and their structure-activity relationship, it is highly probable that the hydrogen atom transfer (HAT) mechanism, along with SET, play crucial roles in antioxidant reaction mechanisms.

As indicated by the TLC–FRAP assay, the analyzed terpenes did not exhibit high antioxidant activity. Indeed, only three (p-cymene, eucalyptol, and γ-terpinene) turned out to be more active in the FRAP–Tween 20 solution analysis. The main problem in the determination of the reducing activity of terpenes via TLC–FRAP assay is their low water solubility. While the terpenes did not indicate the expected activity in the test, gallic acid (a standard antioxidant) appeared as a dark spot on a light background, demonstrating that combining FRAP analysis with TLC is possible. Taking into account the good hydrosolubility of alcoholic extracts, the combination of TLC and FRAP could be used as a fast method to determine the reducing activity of extracts and ascertain their most active ingredients. 

## 4. Materials and Methods

### 4.1. Materials

The terpenes (−)-isopulegol (≥99%), menthol (≥99%), p-cymene (≥99%), eucalyptol (≥99%), (R)-(+)-pulegone (97%), γ-terpinene (97%), α-terpinene (≥95%), linalool (≥97%), (S)-(+)-carvone (≥96%), citronellal (≥95%), (−)-terpinene-4-ol (≥95%), citral (≥95%), menthone (≥90%), farnesene (mixtures of isomers, ≥90%), α-phellandrene (≥90%), β-myrcene (≥90%), and TPTZ (2,4,6-Tri(2-pyridyl)-s-triazine)) were obtained from Sigma-Aldrich (St. Louis, MO, USA). Acetic acid (ACS), FeCl_3_ × 6H_2_O, FeSO_4_ × 7H_2_O, phosphoric acid (ACS), hydrochloric acid (ACS), chloroform (ACS), ethyl acetate (ACS), 2-butanone (ACS), and methanol (analytic purity grade) were obtained from Polish Reagents (Gliwice, Poland). The standards trolox ((±)-6-hydroxy-2,5,7,8-tetramethylchromane-2-carboxylic acid, 97%) and gallic acid (>98%) were purchased from Sigma-Aldrich (St. Louis, MO, USA).

### 4.2. Methods

#### 4.2.1. Basic FRAP Assay Methodology with Some Modifications

FRAP solution was prepared freshly each time by mixing (10:1:1 *v*/*v*/*v*) 0.3 M acetate buffer (pH 3.6) and 0.01 M TPTZ in 0.04 M HCl and 0.02 M FeCl_3_ × 6H_2_O and kept in the dark. Appropriate amounts of studied terpenes (final concentration of 0.1 M) were dissolved in a calculated small volume of methanol and then mixed with 2.4 mL of FRAP solution. The final volume of mixture was 2.8 mL. The prepared samples were vortexed and incubated at 37 °C by 20 min away from light. Absorbance was then measured at 593 nm using a Genesys 20 UV−vis spectrophotometer (Thermo FisherScientific, Waltham, MA, USA) in a 1 cm quartz cell. FRAP working solution with deionized water instead of a sample was used as a blank. All measurements were carried out in triplicate. The results were calculated into µg·mL^−1^ Fe^2+^ on the calibration curve, which was prepared analogically using an aqueous solution of FeSO_4_ at the concentration 1–5 µg·mL^−1^ (y = 0.311x + 0.0514, R^2^ = 0.9965). Additionally, trolox and gallic acid were used as standards. 

The assay was modified in order to determine the most beneficial reaction conditions for terpenes.

(a) Preparation of a terpene–methanol mixture that was added to the basic FRAP solution.
(b) Replacement of methanol (to terpenes dissolution) with other solvents (chloroform, ethyl acetate, or 2-butanone), in which terpenes revealed higher antioxidant activity in previous studies [12].
(c) Addition of Tween 20: The studied terpenes revealed weak solubility in aqueous conditions, which significantly influenced their reducing activity. Additionally, despite the blue color of the samples, the obtained results were falsified due to opacification. In order to resolve this problem, Tween 20 was added to the FRAP solution. The amount of the surfactant was kept below critical micelle concentration (CMC), which, for Tween 20, is equal 0.06 mM. The assay was conducted in two mixing orders, i.e., (1) mixing terpene and methanol, then FRAP and Tween 20, and (2) FRAP and Tween 20, then adding terpene and methanol to 2.8 mL.

#### 4.2.2. FRAP-TLC

The experiment was performed with use of chromatographic HPTLC silica gel 60 (F254) plates (10 cm × 5 cm). Firstly, the studied terpenes were dissolved in methanol in order to facilitate application of the compounds onto the plates. In the first step, appropriate volumes of terpenes and gallic acid solution (as a standard compound), corresponding to 0.1 mg of each compound, were applied onto the plates. Afterward, the plates were sprayed with FRAP solution in a ratio of 10:2:2 (acetate buffer:FeCl_3_:TPTZ). The plates were then scanned at time 0 and after 20 min. In the second step, the procedure was analogous to the first, but the FRAP solution was enriched with Tween 20 addition. The amount of the surfactant was kept below CMC. Active compounds were identified as blue spots on a white background.

## 5. Conclusions

The presented study results revealed that the most promising optimization for FRAP assay among those proposed was augmentation with surfactant. In this experiment, activity changes were observed both through spectrophotometric analysis, as well as by TLC–FRAP assay. In the case of spectrophotometric assay, only two terpenes (isopulegol and linalool) demonstrated higher reducing activities compared to the standard, but the positive influence of surfactant was observed by reduced clouding in the studied solutions, as well as by a reduced false-positive effect (e.g., α-terpinene, α-phellandrene). Despite the solution not showing a spectacular impact on antioxidant activity improvement, the obtained results were more reliable and reproducible. Similarly, the positive effect of Tween 20 was observed for the TLC–FRAP procedure, where adding surfactant to the FRAP solution brought about a situation wherein the active compounds appeared as dark spots in comparison to the nonmodified assay. Moreover, through Tween 20 addition, it was evident that γ-terpinene was active in comparison to the nonmodified assay where the terpene was observed to be inactive. Additionally, the obtained results allowed better insight into the mechanism of antioxidation and confirmed the significant influence of conjugated double bonds in the free radical scavenger and metal ion reduction process. Considering the antioxidant and AChE inhibitory activities of the analyzed terpenes, as well as their Fe(III) reduction abilities, the compounds could be considered as possible anti-neurodegenerative agents. However, more investigation is necessary.

## Figures and Tables

**Table 1:** Ferris-reduced antioxidant power (FRAP) units, trolox equivalent, and gallic acid equivalent obtained for a selected group of terpenes during nonmodified FRAP assay.

Terpenes	FRAP Units (Fe^2+^ µg·mL^−1^)		Trolox Equivalent (µg·mL^−1^)		Gallic Acid Equivalent (µg·mL^−1^)	
	0.1 M	1 mg·mL^−1^	0.1 M	1 mg·mL^−1^	0.1 M	1 mg·mL^−1^
*γ*-terpinene *	-	4.97	-	11.34	-	1.59
citral	3.45	4.30	7.93	9.85	1.10	1.38
carvone	3.58	0.44	8.24	1.19	1.15	1.13
*α*-phellandrene	False-positive effect					
*α*-terpinene	False-positive effect					
*α*-pinene	clouding/lack of color					
farnesene	clouding/lack of color					
eucalyptol	clouding/lack of color					
terpinene-4-ol	clouding/lack of color					
p-cymene	clouding/lack of color					
linalool	clouding/lack of color					
β-myrcene	clouding/lack of color					
citronellal	clouding/lack of color					
isopulegol	clouding/lack of color					
menthol	clouding/lack of color					

* Due to the high activity of γ-terpinene, the assay was determined for a concentration of 0.5 mg·mL^−1^. In the case of a concentration of 0.1 M, absorbance was >>3.400.

**Table 2:** FRAP units, trolox equivalent, and gallic acid equivalent obtained for a selected group of terpenes via FRAP assay modified by the addition of Tween 20.

Terpenes	FRAP Units (Fe^2+^ µg·mL^−1^)		Trolox Equivalent (µg·mL^−1^)		Gallic Acid Equivalent (µg·mL^−1^)	
	0.1 M	1 mg·mL^−1^	0.1 M	1 mg·mL^−1^	0.1 M	1 mg·mL^−1^
γ-terpinene *	-	1.38 (for 0.25 mg·mL^−1^)	-	7.51	-	1.04
citral	3.21	1.04	7.43	2.52	1.03	0.32
Carvone **	False-positive results	0.28	False-positive results	0.83	False-positive results	0.08
α-phellandrene	2.47	1.45	5.74	3.45	0.79	0.45
α-terpinene ***	-	5.75 (for 0.1 mg·mL^−1^)	-	13.09		1.84
isopulegol	0.38	0.01	1.06	0.05	0.11	0.01
linalool	0.34	0.09	0.96	0.40	0.09	0.01
α-pinene	False-positive results ****					
farnesene	clouding/lack of color					
eucalyptol	clouding/lack of color					
terpinene-4-ol	clouding/lack of color					
p-cymene	clouding/lack of color					
β-myrcene	clouding/lack of color					
citronellal	clouding/lack of color					
menthol	clouding/lack of color					

* γ-Terpinene: This terpene revealed high activity at a concentration of 0.1 M and 1 mg·mL^−^^1^, for which absorbance was >3.000. The measurements were performed at a concentration of 0.25 mg·mL^−^^1^. ** Carvone: Measurements were performed at a concentration of 0.5 mg·mL^−^^1^. *** α-Terpinene: This terpene exhibited high activity at a concentration of 0.1 M and 1 mg·mL^−^^1^, for which absorbance was >3.000. The measurements were performed at a concentration of 0.1 mg·mL^−^^1^. **** False-positive results: This effect was observed for probes with the blue color but clouding appeared simultaneously.
